# Origins of Systems Biology in William Harvey’s Masterpiece on the Movement of the Heart and the Blood in Animals

**DOI:** 10.3390/ijms10041658

**Published:** 2009-04-17

**Authors:** Charles Auffray, Denis Noble

**Affiliations:** 1 Functional Genomics and Systems Biology for Health, CNRS Institute of Biological Sciences - 7, rue Guy Moquet, BP8, 94801 Villejuif, France; 2 Department of Physiology, Anatomy and Genetics, Balliol College, Oxford University, Parks Road, Oxford OX1 3PT, United Kingdom. E-Mail: denis.noble@dpag.ox.ac.uk

**Keywords:** William Harvey, heart rhythm, circulation of the blood, mathematical deduction: experimental verification, systems biology

## Abstract

In this article we continue our exploration of the historical roots of systems biology by considering the work of William Harvey. Central arguments in his work on the movement of the heart and the circulation of the blood can be shown to presage the concepts and methods of integrative systems biology. These include: (a) the analysis of the level of biological organization at which a function (e.g. cardiac rhythm) can be said to occur; (b) the use of quantitative mathematical modelling to generate testable hypotheses and deduce a fundamental physiological principle (the circulation of the blood) and (c) the iterative submission of his predictions to an experimental test. This article is the result of a tri-lingual study: as Harvey’s masterpiece was published in Latin in 1628, we have checked the original edition and compared it with and between the English and French translations, some of which are given as notes to inform the reader of differences in interpretation.

## 1. Introduction

In recent articles, we have both drawn attention to some of the historical antecedents of modern systems biology, notably in articles referring to Claude Bernard’s *Introduction à l’étude de la Médicine Expérimentale* [1] and to Gregor Mendel’s *Versuche über Pflanzen-Hybriden*, [2] both published in 1865. The first is considered as the founder of modern experimental medicine, while the second laid the ground for the development of genetics. We argued that both approached and unraveled the functioning of the biological systems they were studying through a highly relevant combination of experiment and modelling which is the hallmark of systems biology. In this article we draw attention to the very important historical antecedent represented by the work of William Harvey (1578–1657). While there may be no generally accepted and simple definition of systems biology, many good expressions of its main features can be found in review articles and books ([3–18] see also the paper by Saks *et al*. in this issue [19]), and it is almost universal to refer in some way to the concept of level and to the role of mathematics, whether they are combined in data-driven (bottom-up) or top-down (model-driven) approaches, or the middle-out (question-driven) research strategy that we favor. These two features appear prominently in the masterpiece of William Harvey, *Exercitatio anatomica de motu cordis et sanguinis in animalibus* published in Latin in Frankfurt in 1628 (translated into modern English by Gweneth Whitteridge [20], and into French by Charles Richet [21]), where he reported his experimental work demonstrating the circulation of blood in animals. Identifying historical precedents for modern biological ideas and methods is important. A subject that neglects its roots fails to benefit from the insights and problems of our predecessors. It is also somewhat humbling to realize that, however enthusiastic we may be about the modern systems approach to biology, the approach is not entirely new. Moreover, claiming such antecedents as Harvey, Bernard and Mendel serves to encourage other biologists to view systems biology in a more favourable light.

## 2. Results and Discussion

### 2.1. Identifying the biological level at which rhythm is generated and integrated

Harvey first describes an experiment in which he seeks a lower level than the organ for the origin of the rhythmic activity of the heart. He writes:

“The heart of an eel and of certain other fish and animals, having been taken out of the body, beats without auricles. Furthermore, if you cut it in pieces, you will see the separate pieces each contract and relax, so that in them the very body of the heart beats and leaps after the auricles have ceased to move.”

Harvey could not, in his day, take this dissection further down to discover that the rhythmic mechanism was integrated at the level of individual cells (see [22], chapter 5), since the cell theory was formulated by Matthias Schleiden (1804–1881) and Theodor Schwann (1810–1882) two centuries later based on observations with the microscope introduced in practice in the life sciences by Anton van Leeuwenhoek (1632–1723) only after Harvey’s death. However, he was the first to realise that rhythmicity was a property of the smallest structures he could discern.

### 2.2. Demonstration of the circulation of the blood through a systems approach

The discovery for which Harvey is best-known is, of course, the circulation of the blood. Already in 1616, in his lecture notes *Prelectiones Anatomiae Universalis* [23], he wrote:

“So it is proved that a continual movement of the blood in a circle is caused by the beat of the heart.”

It is perhaps less well-known that this was the result of a careful series of observations and calculations subjected to an iterative process of modelling and experimental validation which has already all the features of a systems biology inquiry which typically comprises the following steps: formulate a general or specific question; define the components of a biological system and collect previous relevant datasets; integrate them to formulate an initial model of the system and generate testable predictions and hypotheses; systematically perturb the components of the system experimentally or through simulation, and study the results; compare the responses observed to those predicted by the model; refine the model so that its prediction fit best to the experimental observations; conceive and test new experimental perturbations to distinguish between the multiple competing hypotheses; iterate the process until a suitable response to the initial question is obtained [9, 13]. In what follows, we examine how William Harvey goes through this multi-step process to address the general and fundamental question of the significance of the movements of the heart and the blood for the understanding of life and disease in animals.

#### 2.2.1. Critical assessment of previous data

The introduction and the first seven chapters of his book are devoted to a critical assessment of previous work by the Greek philosophers and naturalists considered as the fathers of medicine: Hippocrates of Cos (460 BC-370 BC), Aristotle (384 BC- 322 BC), Eristratus of Chios (304 BC- 250 BC), and Galen of Pergamum (129–200), the founder of the medical practice in use until Harvey’s time.

As a result, he was able to assemble in a coherent manner a wealth of relevant information gathered by his predecessors, identifying uncertainties and contradictions in the description of the movement of the heart and the blood, and dismissing factual errors of observation or interpretation. While he cites them explicitly and extensively, he does not refer to the work of the Arabic polymath Ibn al-Nafis (1213–1288) or of Miguel Servet (1511–1553) who both described the pulmonary circulation independently, but remained unknown to him. Their observations were most likely conveyed by his immediate predecessors the anatomists Andreas Vesalius (1514–1564), who had been in contact with Servet, and Realdo Colombo (1510–1559) as they both worked for some time in Padua where Harvey obtained his medical degree in 1602. Details on the life of Harvey can be found in Keynes [24] and Whitteridge [25], while his anatomical lectures have been completely translated in a bilingual (Latin and English) edition [23].

While in Padua, Harvey was influenced by Girolamo Fabrizi d’Acquapendente or Fabricius (1537–1619) his teacher at the Faculty of medicine, and became fascinated by the valves of the veins (already known to Erasistratus). He showed that these could pass the blood only in one direction. From which it followed that the blood that was taken out to the limbs and organs by the arteries had to return via the veins. The existence of the valves ensured that it could not be just an ebb and flow of fluid, as had been taught since antiquity.

#### 2.2.2. Formulation of a model and derivation of testable hypotheses

In chapter eight of *Exercitatio anatomica de motu cordis et sanguinis in animalibus* (*Decopia sanguinis transeuntis per cor e venis in arteria, et de circulari motu sanguinis* [26]), Harvey presents his model: the circular movement of blood (*de circolari motu sanguinis*) depends on the movements and pulsations of the heart. The model is based on a quantitative evaluation of the amount of blood passing through the heart, the veins and the arteries, and the disposition of the valves in the heart and the blood vessels.

In chapter nine (*Esse sanguinis circuitum ex primo supposito confirmato* [27]), he derives three hypotheses (*suppositio*) from his model, which he intends to demonstrate through experiments: in a brief period of time, the totality of the blood in the organism passes 1) from the veins into the arteries; 2) from the arteries in all body parts; and 3) from the body parts to the heart through the veins. He states:

“These proved, I think it will be clear that the blood circulates, passing away from the heart to the extremities and then returning back to the heart, thus moving in a circle”[28]

#### 2.2.3. Quantitative assessment of experimental parameters

He then proceeds with a numerical calculation, based on a quantitative estimation of the parameters and of their variation: the volume of blood in the heart, the volume of blood ejected from the left ventricle into the aorta at each contraction, the number of contractions in half an hour or a day. He writes:

“Then we may suppose in man that a single heart beat would force out either a half ounce, three drams, or even one dram of blood, which because the valvular block could not flow back that way into the heart. The heart makes more than a thousand beats in half an hour, in some two, three, or even four thousand. Multiplying by the drams, there will be in half an hour either 3,000 drams, 2,000 drams, five hundred ounces, or some other such proportionate amount of blood forced into the arteries by the heart, but always a greater quantity than is present in the whole body.”[29]

and concludes:

“On this assumption of the passage of blood, made as a basis for argument, and from the estimation of the pulse rate, it is apparent that the entire quantity of blood passes from the veins to the arteries through the heart, and likewise through the lungs.”[30]

and

“But suppose even the smallest amount of blood be transmitted through the lungs and heart at a single beat, a greater quantity would eventually be pumped into the arteries and the body than could be furnished by the food consumed, unless by constantly making a circuit and returning.” [31]

#### 2.2.4. Submission of the mathematical predictions to experimental tests

Without the mathematics, the conclusion would not have been reached. But Harvey went even further. From the prediction of his calculation he proceeds to the key experiment. The calculation predicts that the body should empty itself of blood in half an hour if the blood is prevented from circulating:

“This is also clearly to be seen by any who watch the dissection of living creatures, for not only if the great artery be cut, but, as Galen proves, even in man himself, if any artery even the smallest be cut, in the space of about half an hour, the whole mass of blood will be drained out of the whole body…”

This is the iteration between theory and experiment that is essential to success of a systems approach today as it was already in Harvey’s time.

He summarizes at the beginning of chapter ten (*Primum suppositum decopia pertranseuntis sanguinis e venis in arterias, et esse sanguinis circuitum ab obiectionibus vindicatur, et experimentis ulterius confirmatur* [32]):

“Whether the matter be referred to calculation or to experiment and dissection, the important proposition has been established that blood is continually poured into the arteries in a greater amount than can be supplied by the food. Since it all flows past in so short a time, it must be made to flow in a circle.”[33]

He then proceeds in a similar manner in chapters eleven (*Secundum suppositum confirmatur* [34]) and twelve (*Esse sanguinis circuitum ex secundo supposito confirmato* [35]) to demonstrate the second hypothesis, on the basis of observations and numerical calculations performed during experiments using ligatures and compressions, discussing their consequences in terms of medical practice:

“If these things are so, we may very readily compute the amount of blood and come to some conclusion on its circular motion.”[36]

In chapter thirteen (*Tertium suppositum confirmatur, et esse sanguinis circuitum ex tertio supposito* [37]), Harvey endeavours to prove the third hypothesis:

“This proposition will be perfectly clear from a consideration of the valves found in the venous cavities, from their functions, and from experiments demonstrable with them.”

He bases his argument on a series of experiments in which he details the consequences of ligatures and compressions exerted on arm veins, as illustrated in anatomic schemas, and supported once again by a numerical calculation:

“By careful reckoning, of course, the quantity of blood forced up beyond the valve by a single compression may be estimated, and this multiplied by a thousand gives so much blood transmitted in this way through a single portion of the veins in a relatively short time, that without doubt you will be very easily convinced by the quickness of its passage of the circulation of the blood.”[38]

He concludes briefly in chapter fourteen (*Conclusio demonstrationis de sanguinis circuitu* [39]) on the demonstration of the circulation of blood, ending the first iteration of a typical systems biology approach:

“It must therefore be concluded that the blood in the animal body moves around in a circle continuously, and that the action or function of the heart is to accomplish this by pumping. This is the only reason for the motion and beat of the heart.”[40]

#### 2.2.5. Refinement of the model through further observations

The last three chapters are the beginning of a second iteration, intended to confirm circulation on the basis of compatible physiological observations (chapter fifteen: *Sanguinis circuitus rationibus verisimilibus confirmatur* [41]), the consequences for the treatment of diseases (chapter sixteen: *Sanguinis circuitus ex consequentibus probatur* [42]):

“Assuming the truth of this proposition there are certain consequences which are useful in coaxing belief a posteriori. Although some of them may seem to be clouded in considerable doubt, a reasonable case may easily be made of them.”[43]

and finally a number of anatomical observations on the structure and development of the heart in diverse animals (chapter seventeen: *Confirmatur sanguinis motus, et circuitus ex apparentibus Corde, et ex iis, quaex dissectione Anatomicapatent* [44]).

### 2.3. Circulation, circuit and capillaries

While the central notion of circular movement is explicit from Chapter eight on, it is worth pointing that Harvey made a clear distinction between the anatomical structures and the action taking place within them (the movement of the heart and the circulation of the blood). This is apparent in his repeated use in eight of the last nine chapter headings of the Latin word *circuitus*, which has been translated rather loosely as “circulation” in both the English and French versions. As an outstanding anatomist, he was well aware that in order to allow “circulation” of the blood, the “circuit” had to be closed at the juncture between the arteries and the veins. It is therefore worth pointing to his reference to “*venis capillaribus in paruas ramifications*” (chapter fifteen), and “*ultimae diuisiones capillares, arteriolae videantur*” (chapter seventeen) which were wrongly translated as “tiny veins” and “terminal arteries” [20], giving the impression he had missed this important notion. The existence and role of capillaries in the circulation would be demonstrated only later in 1661 by the Italian histologist Marcello Malpighi (1628–1694) when he examined blood vessels in frogs using the then recently invented microscope. Malpighi is also famous for giving his name to a number of anatomical structures in animals and insects, and was the first to report his experimental findings in scientific articles including a method section, as has become the routine practice in modern science ever since.

## 3. Conclusions: Harvey and the Conceptual and Ethical Foundations of Modern Science

Harvey’s achievements are all the more remarkable since they were performed when the basic concepts and methods that would form the bases for the development of modern science were just being established. Francis Bacon (1561–1626) published his *Novum Organum* in 1620, and René Descartes (1596–1650) *Discours de la Méthode* in 1637, the same year when he introduced the algebraic notation using Latin letters. It is therefore not surprising that all of Harvey’s calculations are expressed literally. Despite these limitations, he was very much aware of the conceptual and practical aspects of his experiments, which were known to Descartes himself, as is shown in his responses to the criticisms of Jean Riolan Fils, of Paris, the chief medical doctor of Louis XIII’s mother (Harvey himself was the doctor of two kings of England, James I and Charles I). Riolan, one of Harvey’s severest critics on the circulation of the blood wrote in his *Encheiridium anatomicum et patholigicum*:

“That this circulatory movement may be more easily and more conveniently maintained, William Harvey, Englishman, Royal Physician, and author and discoverer of this movement of the blood, and John Waleus, professor of Leyden, who defends and vigorously upholds it, believe the blood to be taken through the lungs from the right to the left ventricle of the heart and deny its passage through the septum of the heart, and so they believe that in one or two hours all the blood passes through the heart and through the whole body. This I do not admit.”

Riolan was trying, valiantly but vainly, to reconcile strict Galenic teaching with Harvey’s observations. “The resulting inconsistencies and contradictions Harvey was not slow to point out” [25]. In his *Exercitationes duae anatomicae de circulatione sanguinis* (Two anatomical exercises concerning the circulation of blood) [21, 24], published in 1649 in response to Riolan, Harvey states:

“There is no science that derives only from a priori ideas, and there is no solid and certain knowledge that does not taken its origin from our sense organs” (first dissertation) [45].

“But it is our senses, not accepted theories, dissection and not the dreams of imagination, that should teach us what is true or false (second dissertation) [46]

“A man remarkable for his brilliant genius, René Descartes, who I thank for the complimentary reference that he has made of me” (second dissertation) [47].

“But I think it a thing unworthy of a Philosopher and a searcher of the truth, to return bad words for bad words; and I think I shall do better and more advised, if with the light of true and evident observations I shall wipe away those symptoms of incivility” (second dissertation) [48].

Only two years later, using the same approach as for the study of circulation, he published *Exercitationes de Generatione Animalium* [49] which contributed to the foundation of modern embryology. It would be interesting to speculate on why some of the important features of the systems approach, particularly the use of mathematics and modeling, became neglected until recently. Factors that may have played a part include: the sheer difficulty of applying mathematics in biology; the lack of suitable means for solving the problems, which became tractable only after the invention of the digital computer in the second half of the 20^th^ century; the rise of a positivist (reductionist) bias in biology from the 19^th^ century onwards (many leading physiological journals actually excluded mathematical biology); and, most recently, the rise of molecular biology, with a tendency to avoid theory (except, very significantly, the central dogma of molecular biology). A full treatment of these and other factors would require a detailed historical analysis and will be the subject of a further article.

In any event, almost four centuries after he published his masterpiece, the concepts and experimental principles that were laid out by William Harvey are some of the pillars on which several branches of natural and engineering sciences have been flourishing. This common origin should facilitate the cross-fertilization of biology, following the quantitative footsteps of physics and engineering, thus enabling the extension of physiology into integrative systems biology.

## Figures and Tables

**Figure 1.**
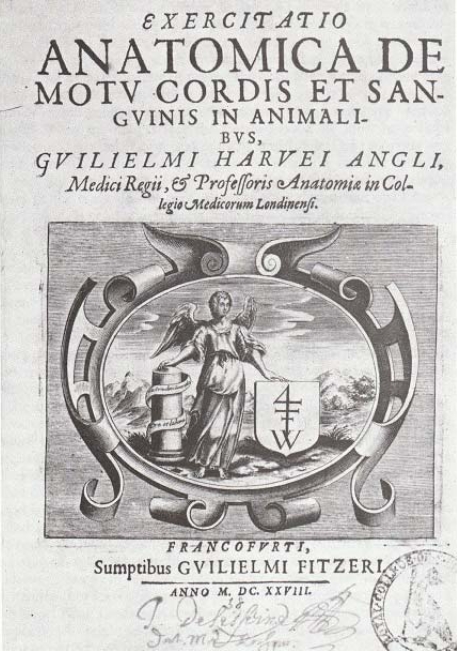
Title page of *Exercitatio anatomica de motu cordis et sanguinis in animalibus*, 1628 [20].

## References

[1] Noble D (2008). Claude Bernard, the first systems biologist, and the future of physiology. Exp Physiol.

[2] Aux sources de la biologie des systèmes et de la génétique: la pertinence des expérimentations de Gregor Mendel sur le développement des plantes hybrides [In French]. http://www.ircm.qc.ca/bioethique/obsgenetique/, published 2005, accessed March 15, 2009.

[3] Szallasi Z, Stelling J, Periwal V (2006). System Modeling in Cellular Biology: From Concepts to Nuts and Bolt.

[4] Palsson B (2006). Systems Biology: Properties of Reconstructed Network.

[5] Kaneko K (2006). Life: An Introduction to Complex Systems Biology;.

[6] Alon U (2006). An Introduction to Systems Biology: Design Principles of Biological Circuits;.

[7] Klipp E, Herwig R, Kowald A, Wierling C, Lehrach H (2005). Systems Biology in Practice. Concepts, Implementation and Application.

[8] Alberghina L, Westerhoff HV (2005). Systems Biology: Definitions and Perspective.

[9] Ideker T, Galitski T, Hood L (2001). A new approach to decoding life: systems biology. Annu. Rev. Genomics Hum. Genet.

[10] Wolkenhauer O (2001). Systems biology: The reincarnation of systems theory applied in biology. Brief Bioinform.

[11] Kitano H (2002). Systems biology: a brief overview. Science.

[12] Noble D (2002). Modeling the heart—from genes to cells to the whole organ. Science.

[13] Auffray C, Imbeaud S, Oux-Rouquie M, Hood L (2003). From functional genomics to systems biology: Concepts and practices. C R Biol.

[14] Nicholson JK, Holmes E, Lindon JC, Wilson ID (2004). The challenges of modeling mammalian biocomplexity. Nat Biotechnol.

[15] Westerhoff HV, Palsson BO (2004). The evolution of molecular biology into systems biology. Nat Biotechnol.

[16] Auffray C, Nottale L (2008). Scale relativity theory and integrative systems biology: 1. Founding principles and scale laws. Prog Biophys Mol Biol.

[17] Auffray C, Chen Z, Hood L (2009). Systems medicine: The future of medical genomics and healthcare. Genome Med.

[18] Kitano H (2001). Foundations of Systems Biology;.

[19] Saks V, Monge C, Guzun R (2009). Philosophical basis and some historical aspects of systems biology: From Hegel to Noble - Applications for bioenergetics research. Int J Mol Sci.

[20] Harvey W (1976). An Anatomical Disputation Concerning the Movement of the Heart and Blood in Living Creatures.

[21] Harvey W (1872). La circulation du sang Des mouvements du cœur chez l'Homme et les animaux Deux réponses à Riolan [In French].

[22] Noble D (2006). The Music of Life.

[23] Whitteridge G (1964). The Anatomical Lectures of William Harvey.

[24] Keynes G (1978). The Life of William Harvey.

[25] Whitteridge G (1971). William, Harvey and the Circulation of the Blood [In Latin and English].

[26] Amount of blood passing through the heart from the veins to the arteries, and the circular motion of the blood [20]; De la quantité de sang qui passe par le coeur, des veines dans les artères, et du mouvement circulaire du sang [21].

[27] The circulation of the blood is proved by a prime consideration [20]; Démonstration de la circulation du sang par la confirmation de la première hypothèse [21].

[28] « Je dis qu’alors évidemment le sang circule, qu’il est chassé du coeur aux extrémités, et qu’il revient des extrémités au coeur, et ainsi de suite, accomplissant un mouvement circulaire. » [21].

[29] The Apothecaries of Troy weight is used: 3 scruples equal 1 dram; 8 drams equal 1 ounce; 12 ounces equal 1 pound. This was in general use in Europe (Note from Leake, CD in the tercentennial edition of Harvey's *Exercitatio anatomica de motu cordis et sanguinis in animalibus*, Springfield and Baltimore: Thomas, C.C., ed., 1928).

[30] « Ainsi en supputant la quantité de sang que le coeur envoie à chaque contraction et en comptant ces contractions, on voit que toute la masse du sang passe des veines dans les artères par le coeur et aussi par les poumons. » [21].

[31] « Mais, quelque petite que soit la quantité de sang qui passe par le coeur et les poumons, il y en a néanmoins bien trop pour que les aliments ingérés y puissent suffire, à moins que le sang ne revienne par les mêmes trajets. » [21].

[32] The first proposition, concerning the amount of blood passing from veins to arteries, during the circulation of the blood, is freed from objections, and confirmed by experiments [20]; La première hypothèse sur la circulation du sang, fondée sur la quantité de sang qui passe des veines dans les artères, est confirmée par des expériences ; et les objections qu’on lui avaient opposées sont réfutées [21].

[33] « Jusqu’ici le calcul, les expériences, les dissections ont confirmé notre première hypothèse, que le sang passe continuellement dans les artères, et en trop grande quantité pour que les aliments y puissent suffire, en sorte que comme la totalité du sang passe en très peu de temps par le même endroit, le sang doit nécessairement revenir par les mêmes voies et accomplit un véritable circuit. » [21].

[34] The second proposition is proven [20]; Confirmation de la seconde hypothèse [21].

[35] That there is a circulation of the blood follows from the proof of the second proposition [20]; La confirmation de la seconde hypothèse démontre la circulation du sang [21].

[36] « Maintenant calculons la quantité de sang qui passe par les veines, et démontrons à l’aide de calculs le mouvement circulaire du sang. » [21].

[37] The third proposition is proven, and the circulation of the blood is demonstrated from it [20]; Confirmation de la troisième hypothèse, qui démontre la circulation du sang [21].

[38] « Calculez maintenant combien de sang vous aurez arrêté en mettant le doigt au-dessus de la valvule, et multipliez cette quantité par milliers ; vous verrez alors quelle grande quantité de sang passe ainsi dans cette petite portion de veine, en un temps aussi court, et je crois que vous serez bien convaincu de la circulation du sang et de la rapidité de son mouvement » [21].

[39] Conclusion on the demonstration of the circulation of blood [20]; Conclusion de la démonstration de la circulation du sang [21].

[40] « Il faut donc nécessairement conclure que chez les animaux le sang est animé d’un mouvement circulaire qui l’emporte dans une agitation perpétuelle, et que c’est là le rôle, c’est là la fonction du coeur, dont la contraction est la cause unique de tous ces mouvements. » [21].

[41] The circulation of blood is confirmed by plausible methods [20]; La circulation du sang confirmée par les vraisemblances.

[42] The circulation of the blood is supported by its implications [20]; La circulation du sang prouvée par les implications qu’elle entraîne [21].

[43] « Il y a encore des problèmes qui sont comme la conséquence de la vérité de la circulation. Ils ne sont point inutiles pour y faire croire et leur démonstration est comme un argument a posteriori. » [21].

[44] The motion and circulation of the blood is established by what is displayed in the heart and elsewhere by anatomical investigation [20]; Confirmation du mouvement et de la circulation du sang par ce que nous voyons dans le Coeur, et par les observations anatomiques [21].

[45] « Il n’y a pas de science qui ne dérive d’une idée a priori, et il n’y a pas de connaissance solide et sûre qui ne tire son origine des sens. » [21].

[46] « Or ce sont nos sens et non les théories admises, la dissection et non les rêves de l’imagination qui doivent nous apprendre si elles sont vraies ou fausses.» [21].

[47] « Un homme remarquable par son brillant génie, René Descartes, que je remercie de la mention élogieuse qu’il a fait de moi. » [21].

[48] « Pour moi, je trouve que répondre à des injures par des injures est une action indigne d’un philosophe qui cherche la vérité, et qu’il vaut mieux confondre ces méchants par la lumière de l’observation et de la vérité.» [21].

[49] Whitteridge G (1981). Disputations touching the Generation of Animals;.

